# Can non-swallowing function assessment predict nasogastric tube removal in patients with poststroke dysphagia? A clinical study

**DOI:** 10.3389/fneur.2022.984707

**Published:** 2023-03-01

**Authors:** Bingjie Li, Tong Zhang, Jun Zhao, Pengkun Li, Zhangwei Wu, Shengjie Zhao

**Affiliations:** Department of Neurology, Capital Medical University School of Rehabilitation Medicine, China Rehabilitation Research Center, Beijing, China

**Keywords:** dysphagia, stroke, prognosis, functional improvement, NIHSS

## Abstract

**Objective:**

This study aimed to predict nasogastric tube (NGT) removal in patients with poststroke dysphagia (PSD) by non-swallowing function assessment.

**Methods:**

We enrolled 232 eligible patients and performed rehabilitation. The Fugl-Meyer assessment motor (FMM) and National Institute of Health Stroke Scale (NIHSS) scores were used to measure the motor and overall nervous system functions. Predictors for NGT removal in patients with PSD after rehabilitation were analyzed.

**Results:**

Of the 232 included patients, the NGTs were removed from 78% of them, while 22% were dependent on a feeding tube after 4 weeks of rehabilitation. Compared to the preserved NGT group, older age, a higher rate of intubation or tracheostomy, and more severe baseline functions were found in the NGT removal group. Age [odds ratio (OR) = 0.907; 95% confidence interval (CI): 0.859–0.957; *p* = 0.000], difference in the FMM score after 4 weeks of rehabilitation (OR = 1.219; 95% CI: 1.145–1.299; *p* = 0.00), and item 9 of NIHSS (OR = 0.488; 95% CI: 0.252–0.946; *p* = 0.034) were predictors of NGT removal after rehabilitation.

**Conclusion:**

We established a predictive model in patients with PSD using a non-swallowing assessment, which enabled us to predict swallowing recovery based on the non-swallowing function.

## Introduction

With advanced treatment for acute stroke and an increased proportion of the elderly in China, stroke survival has improved significantly, and stroke-related disabilities have increased. Poststroke dysphagia (PSD) is a major poststroke disability with a prevalence ranging from 42 to 80%, depending on the evaluation method and the assessment time (1, 2). Some patients with PSD recovered spontaneously in the acute phase, while 11–50% of patients had dysphagia at 6 months (3).

Guidelines for adult stroke rehabilitation and recovery suggested that tube feeds *via* the nasogastric route are reasonable for patients who cannot swallow safely for the first 2–3 weeks after stroke, and percutaneous gastrostomy (PEG) tubes should be placed for patients with a chronic inability to swallow safely (4). The selection criteria for a proper feeding method for patients with PSD in the subacute stage are unclear. However, both methods have limitations. The use of a nasogastric tube (NGT) is convenient, non-invasive, and more likely to result in food reflux, aspiration, and lung infection with long-term indwelling. Despite the benefits of providing nutritional supplies to patients with PSD, PEG is non-invasive. PEG causes severe complications in 3.8–10% of patients during or immediately after gastrostomy, such as bleeding, perforation, and peritonitis (5–7). Ickenstein et al. (8) found that one-third of patients (31.2%; 24/77) underwent PEG removal before discharge and resumed oral diets. Lin et al. (9) reported that 25.9% of the 181 patients with stroke underwent PEG removal at discharge. Kim et al.'s study (10) provided promising results. Even in severe cases of dysphagia secondary to the lateral medullary syndrome, almost all patients ultimately completely recovered from dysphagia. Therefore, prior knowledge of the possibility of resuming oral diets in patients with PSD in the subacute stage would help the clinical decision-making process in determining whether or not NGT feeding should be prolonged to avoid unnecessary and invasive PEG. Predicting outcomes in patients with PSD is also meaningful in supporting counseling for them and their families by individualizing specific recovery trajectories.

Many studies attempted to develop models to predict PSD outcomes. Signs of aspiration in the first 72 h, lesion locations, age, stroke-related medical complications, cognitive function, type of stroke, and National Institute of Health Stroke Scale (NIHSS) score are prognostic variables for swallowing recovery after acute stroke (11–14). Much of the existing literature on predicting PSD outcomes is limited by including patients with acute stroke who are recovering in the spontaneous state, and research on patients with subacute stroke and recovery after rehabilitation has only been found in one study (15). Considering that dysphagia rehabilitation is an effective intervention in swallowing regain and shortening the duration of dysphagia, it should be considered in the prognosis of swallowing recovery for patients with PSD.

Nasogastric tube removal for patients with PSD reflects the sufficient recovery of swallowing to resume oral feeding. Therefore, the main goals of this study were to (1) evaluate whether or not motor improvement was associated with NGT removal in patients with prolonged PSD and (2) establish a model to predict NGT removal in patients with prolonged PSD.

## Materials and methods

### Participants

We performed a retrospective analysis of demographic and clinical data in consecutive patients with stroke at the Department of Neurology in the China Rehabilitation Research Center between January 2012 and December 2021. The study protocol was approved by the Institutional Review Board of the China Rehabilitation Research Center. Demographic and outcome assessment data were extracted from medical records. Moreover, the patients provided informed consent for the use of their data. Stroke was diagnosed based on computed tomography or magnetic resonance imaging findings.

Participants were included if they met the following criteria: (1) age, 18–80 years; (2) history of the first supratentorial ischemic stroke within 3 months; (3) history of PSD; (4) NGT feeding; (5) no history of swallow rehabilitation training before entering our research center; (6) no loss of consciousness; and (7) functional ability assessments at admission and after 4 weeks of rehabilitation. Participants were excluded if they met any of the following criteria: (1) bilateral lesions; (2) severe cardiac dysfunction, such as acute coronary syndrome, myocardial infarction, and heart failure; (3) serious complications that prevented rehabilitation, such as severe pneumonia, pulmonary embolism, liver dysfunction, or renal dialysis; (4) a history of cancer, chronic obstructive pulmonary disease, malnutrition, chronic kidney disease, and mental disorders; (5) an underlying reason for dysphagia before stroke (e.g., multiple sclerosis, Parkinson's disease, dementia, motor neuron disease, or previous head or neck surgery); (6) endotracheal intubation or tracheotomy; and (7) those passed a videofluoroscopic assessment of swallowing (VFSS) test in the first week after admission.

Two senior neurologists evaluated all patients according to the aforementioned inclusion and exclusion criteria.

### Assessment

The following assessments were performed at the China Rehabilitation Research Center.

Swallowing function assessments and criteria for NGT removal: Swallowing function was examined within the first 24 h after admission by the modified water-swallowing test (16) before swallowing rehabilitation. The modified water-swallowing test was conducted by certified and trained nurses. Patients were placed in a seated position and instructed to drink 3 or 5 ml of water *ad libitum* without using a pipette or pausing. The amount of water was gradually increased from 3 or 5 ml to 10, 30, and 60 ml. At 60 ml, patients were instructed to drink as quickly and safely as possible at their own pace. Subsequently, they had 3 or 5 ml of yogurt with a spoon. The method and instructions for increasing the amount of yogurt were the same as those for drinking water. Coughing, regurgitation, laryngeal movement, or decrease in oxygen saturation during swallowing or 10 min after resulted in a failed modified water-swallowing test and the patient was kept nil by mouth. Patients who passed the screening were permitted to undergo VFSS, the gold-standard diagnostic modality for dysphagia. Furthermore, NGT could be removed when patients with PSD passed VFSS.

Immediately after NGT was withdrawn, speech pathologists performed VFSS. After sitting down with their heads horizontally, they were instructed to swallow 5 and 10 ml of liquid and 5 ml of yogurt three times, which were mixed with a barium solution. The following parameters in VF were assessed: bolus transport from the mouth to the pharynx, bolus holding in the oral cavity, velopharyngeal seal, tongue base movement, pharyngeal constriction, laryngeal elevation, upper esophageal sphincter opening, and bolus stasis at pyriform sinus. Aspiration was also assessed. Impaired lip closure, incomplete oral clearance, repeated piecemeal swallowing, incomplete pharyngeal clearance, penetration, and aspiration were observed. If one or more of the aforementioned symptoms occurred, VFSS was judged to have failed.

Motor function and overall function assessments: The motor function was assessed using the Fugl-Meyer assessment motor score (FMM), which is widely used in clinical practice to measure motor impairment. Stroke severity was assessed using the NIHSS, which is a widely established and validated scale to determine stroke severity and an adjunct to predict PSD with moderate sensitivity and specificity (17). The FMM and NIHSS scores of patients with PSD were assessed at admission and after 4 weeks of rehabilitation.

Onset admission interval (OAI) was defined as the time from the onset of stroke symptoms to admission at our center.

### Treatment procedures

All patients with PSD who failed the swallowing screening test and VFSS were engaged in a coordinated, intensive program of stroke swallowing rehabilitation by a speech–language pathologist for 4 weeks. Speech–language–hearing therapists trained the patients in eating and swallowing using indirect strategies, such as oral care, oral articulation exercises, pharynx-cooling stimulation, and balloon dilatation of the upper esophageal sphincter, as needed. In conjunction with indirect training, direct rehabilitation exercises, such as the Shaker head-lift, Masako maneuver, and expiratory muscle strength training, were prescribed whenever possible based on the assessment. Interventions were performed within 48 h after admission. Furthermore, patients participated in 30-min tailor-made conventional swallowing therapy one time a day, 5 days/week, for 4 weeks. NGT removal for patients with PSD was first evaluated by speech therapists based on clinical judgment, followed by VFSS. During periods of daily speech therapist intervention, physical or occupational therapies on weekends were simultaneously added, and speech and cognitive function rehabilitation were performed whenever necessary.

### Variables

We obtained data on a wide range of factors that may contribute to rehabilitation outcomes. The following categories were used to classify 15 variables: (1) sociodemographic data (age at admission and sex; *n* = 2 variables), (2) medical history (hypertension, diabetes mellitus, hyperlipidemia, and coronary heart disease; *n* = 4 variables), (3) computed topographical findings (stroke side and lesion site; *n* = 2 variables), and (4) poststroke factors (etiology, OAI, intubation or tracheostomy, pneumonia during rehabilitation, venous thrombosis, FMM score improvement after rehabilitation, and NIHSS score improvement after rehabilitation; *n* = 7 variables), many of which could predict or influence swallowing outcomes (18, 19).

### Statistical analysis

The study outcome was defined as NGT removal after 4 weeks of rehabilitation. We compared patients with and without NGT removal using an univariate analysis. Continuous variables are expressed as mean ± standard deviation or median (interquartile range) according to the distribution. The Shapiro–Wilk test was used to assess the normality of data distribution. Differences between groups were analyzed using the independent Student's *t*-test for normally distributed variables and the Wilcoxon rank-sum test for non-normally distributed variables. Categorical variables were expressed as frequency (percentage) and were compared using the χ^2^ test. Multivariate logistic regression analyses were used to identify independent risk factors for delirium in non-agenarians after hip fracture surgery. Variables eligible for inclusion in multivariate models include those significant at a *p* < 0.05 in the univariate analyses.

Statistical significance was two-tailed and set at a *p* < 0.05. Except for the evaluation of model performance, performed by R software, statistical analyses were performed using SPSS (version 18.0, Chicago, IL., USA).

## Results

### Subjects characteristics

**Figure 1:**
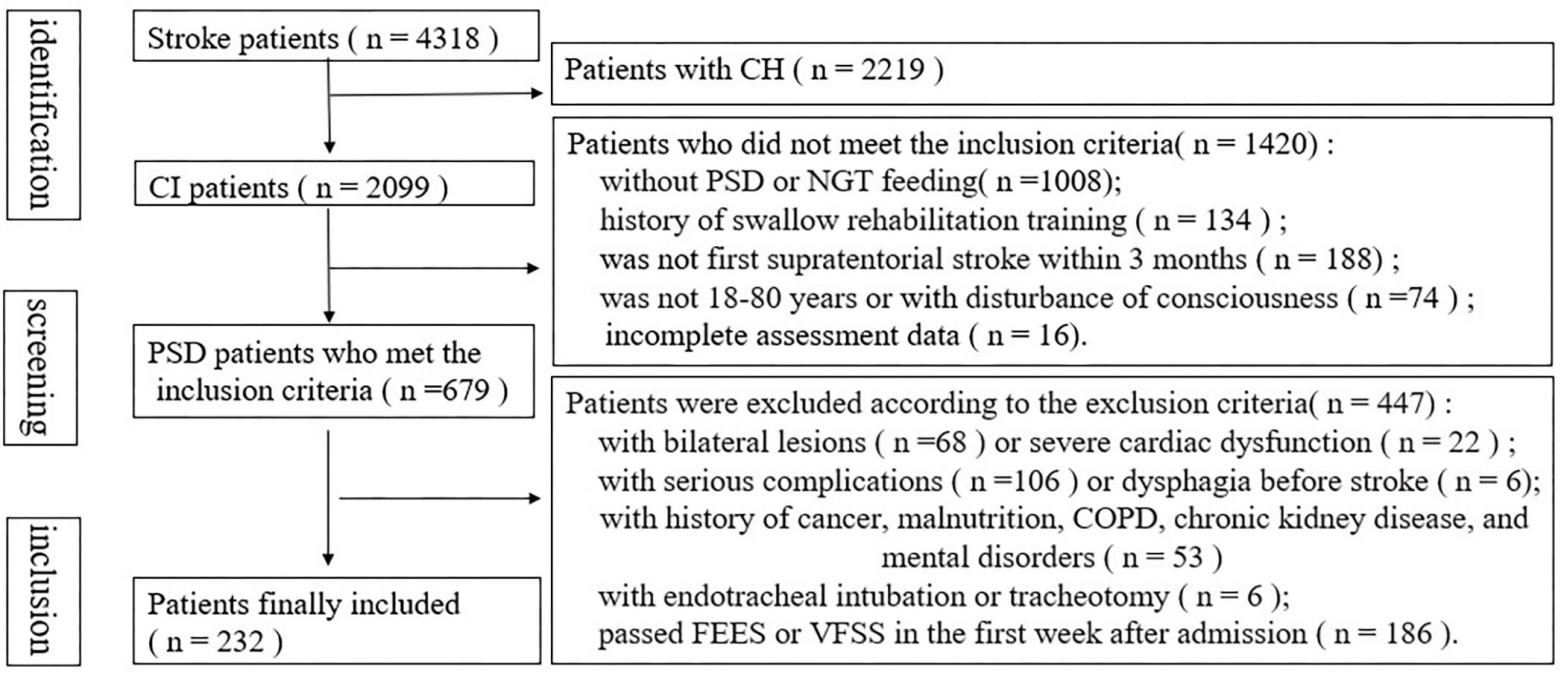
Flow diagram of the selection of patients from our cerebrovascular disease database and specific reasons for exclusion.

A flowchart of the PSD screening from the database of a stroke at our center is presented in Figure 1. Among 4,318 patients with PSD, 679 with NGT were admitted. After excluding patients who met the exclusion criteria, a total of 232 patients were eligible for the study. Notably, 35 patients were completely incapable of oral intake, while others showed one or more of the following findings: impaired lip closure, incomplete oral clearance, repeated piecemeal swallowing, incomplete pharyngeal clearance, penetration, and aspiration.

After 4 weeks of rehabilitation, 181 (78%) of the 232 patients with PSD underwent successful NGT removal. Table 1 shows the clinical characteristics of the study participants. Of the 232 patients included, 74.1% were men, and 52.6% had right hemiplegia. The mean age was 63.5 ± 10.7 years (range, 28–80 years; median 65 years). The average OAI was 28.3 ± 15.7 days (range, 15–90 days; median, 23.5 days). The lesions located at the cortical branch of the middle cerebral artery (MCA), the deep perforating branch of MCA, and the MCA trunk were 21.1, 40.1, and 38.8%, respectively. Patients with PSD experienced low NIHSS and FMM scores at admission. After 4 weeks of rehabilitation, the motor and overall nervous system functions were greatly improved. During rehabilitation, the occurrence of aspiration pneumonia was 20.3%.

**Table 1:** Patients' characteristics.

**Factors**	**Values (*n* = 232)**
Removal of NGT	181 (78%)
Age, years	63.5 ± 10.7
Sex (men), *n* (%)	172 (74.1%)
**Past medical history**	
Hypertension, *n* (%)	169 (72.8%)
Diabetes mellitus, *n* (%)	95 (40.9%)
Dyslipidemia, *n* (%)	144 (62.1%)
Venous thrombosis, *n* (%)	55 (23.7%)
Coronary heart disease, *n* (%)	51 (22.0%)
Onset admission interval (OAI), days	28.8 ± 15.2
Stroke laterality (left), *n* (%)	122 (52.6%)
**Stroke location**	
Cortical branch of MCA	49 (21.1%)
Deep perforating branch of MCA	93 (40.1%)
MCA trunk	90 (38.8%)
**Stroke etiology**	
Large-artery atherosclerosis	149 (64.2%)
Cardioembolic	55 (23.7%)
Small-vessel occlusion	28 (12.1%)
Intubated or tracheostomy, n (%)	17 (7.3%)
Stroke severity (NIHSS) at admission	11.2 ± 4.6
FMM (admission)	24.8 ± 22.9
Pneumonia during rehabilitation, *n* (%)	47 (20.3%)
**Stroke outcome after 1 month**	
FMM (after 1 month)	45.2 ± 25.2
NIHSS score (after 1 month)	5.4 ± 2.2

NGT, nasogastric tube; MCA, middle cerebral artery; NIHSS, National Institute of Health Stroke Scale; FMM, Fugl-Meyer assessment motor score.

Most patients with PSD in our study showed substantial swallowing improvement after rehabilitation: 78% of all 232 included patients underwent NGT removal, while 22% were dependent on feeding tubes at discharge after rehabilitation. Moreover, the literature reports oral feeding recovery in rehabilitation settings in 31–87% of the patients (15).

### Risk factors for NGT removal in patients with PSD

We divided the patients into two groups according to whether or not NGT was removed after 4 weeks of swallowing rehabilitation. Table 2 shows the patient characteristics in the two groups. Except for age, motor function, overall nervous system function, and intubation or tracheostomy, the remaining baseline characteristics were similar between the two groups. Compared to the preserved NGT group, older age, a higher rate of intubation or tracheostomy, and more severe baseline functions were found in the NGT removal group. After rehabilitation, the motor function of the two groups improved greatlywhich were significant in the NGT removal group (Table 2).

**Table 2:** Risk factors for NGT removal in patients with PSD.

	**NGT removed (*N* = 181)**	**NGT reserved (*N* = 51)**	***P*-value**
Age, years	61.7 ± 10.3	70.0 ± 9.6	0.000
Sex (men), *n* (%)	139 (76.8%)	33 (64.7%)	0.082
**Past medical history**			
Hypertension, *n* (%)	134 (74.0%)	35 (68.6%)	0.443
Diabetes mellitus, *n* (%)	70 (38.7%)	25 (49.0%)	0.184
Dyslipidemia, *n* (%)	115 (63.5%)	29 (56.9%)	0.386
Venous thrombosis, *n* (%)	40 (22.1%)	15 (29.4%)	0.278
Coronary heart disease, *n* (%)	41 (22.7%)	10 (19.6%)	0.643
Onset admission interval (OAI), days	28.5 ± 15.6	30.0 ± 13.8	0.523
Stroke laterality (left), *n* (%)	91 (50.3%)	31 (60.8%)	0.184
**Stroke location**			0.163
Cortical branch of MCA	38 (21.0%)	11 (21.6%)	
Deep perforating branch of MCA	78 (43.1%)	15 (29.4%)	
MCA trunk	65 (35.9%)	25 (49.0%)	
**Stroke etiology**			0.234
Large-artery atherosclerosis	112 (61.9%)	37 (72.5%)	
Cardioembolic	44 (24.3%)	11 (21.6%)	
Small-vessel occlusion	25 (13.8%)	3 (5.9%)	
Intubated or tracheostomy	8 (4.4%)	9 (17.6%)	0.001
**Function at admission**			
NIHSS at admission	10.6 ± 4.6	13.3 ± 4.0	0.000
FMM at admission	26.6 ± 23.2	18.4 ± 20.6	0.018
Pneumonia during rehabilitation	39 (21.5%)	8 (15.7%)	0.358
**Stroke outcome after 1 month**			
NIHSS score (after 1 month)	5.1 ± 2.2	6.4 ± 2.1	0.000
FMM (after 1 month)	51.1 ± 23.1	24.6 ± 20.9	0.000
**Functional improvements**			
FMM improvement after rehabilitation	24.5 ± 11.1	6.2 ± 7.6	0.000
NIHSS improvement after rehabilitation	5.5 ± 3.1	6.9 ± 2.7	0.003

PSD, poststroke dysphagia; NGT, nasogastric tube; MCA, middle cerebral artery; NIHSS, National Institute of Health Stroke Scale; FMM, Fugl-Meyer assessment motor score.

### Multivariate analysis for factors that influence NGT removal after rehabilitation in patients with PSD using overall NIHSS scores

The bivariate logistic regression analysis of factors revealed three significant factors as predictors of NGT removal after rehabilitation (Table 3): age [odds ratio (OR) = 0.904; 95% confidence interval (CI): 0.856–0.955; *p* = 0.000], FMM improvement after rehabilitation (OR = 1.241; 95% CI: 1.162–1.326; *p* = 0.000), and NIHSS improvement after rehabilitation (OR = 0.714; 95% CI: 0.590–0.865; *p* = 0.001). Older patients and a lower improvement score could result in limited swallowing functional improvement after inpatient stroke rehabilitation.

**Table 3:** Prediction Model 1—Multivariate analysis for NGT removal after rehabilitation in PSD patients with overall NIHSS.

**Independent variables**	**OR (95% CI)**	**P值**
Age, years	0.904 (0.856–0.955)	0.000
FMM improvement after rehabilitation	1.241 (1.162–1.326)	0.000
NIHSS improvement after rehabilitation	0.714 (0.590–0.865)	0.001
Intubated or tracheostomy	4.516 (0.531–38.373)	0.167

PSD, poststroke dysphagia; NGT, nasogastric tube; NIHSS, National Institute of Health Stroke Scale; FMM, Fugl-Meyer assessment motor score.

### Multivariate analysis of factors that influence NGT removal after rehabilitation in patients with PSD using NIHSS subscores

Table 4 compares the improvements of NIHSS subscores between the two groups to reveal the items playing the leading role in the prediction of recovery. The results suggest that the NGT removal group exhibited improvement scores in the items of blinking eyes and squeezing hands, horizontal extraocular movements, language/aphasia, and dysarthria.

**Table 4:** Risk factors of NIHSS subscores for removal of NGT in patients with PSD.

	**NGT removed *N* = 181**	**NGT reserved *N* = 51**	***P*-value Mann Whitney U test**
1a Level of consciousness	0 (0–0)	0 (0–0)	1.000
1b Ask month and age	0 (0–2)	0 (0–2)	0.448
1c Blink eyes and squeeze hands	0 (0–2)	0 (0–2)	0.005
2 Horizontal extraocular movements	0 (0–2)	0 (0–2)	0.040
3 Visual fields	0 (0–2)	0 (0–2)	0.706
4 Facial palsy	0 (0–2)	0 (0–2)	0.460
5 Arm motor drift	1 (0–3)	2 (0–3)	0.485
6 Leg motor drift	3 (0–3)	3 (0–3)	0.175
7 Limb ataxia	0 (0–1)	0 (0–1)	0.242
8 Sensation	0 (0–2)	0 (0–2)	0.623
9 Language/Aphasia	0 (0–2)	1 (0–2)	0.000
10 Dysarthria	0 (0–1)	0 (0–1)	0.010
11 Extinction/Inattention	0 (0–2)	0 (0–2)	0.831

PSD, poststroke dysphagia; NIHSS, National Institute of Health Stroke Scale.

The multivariate logistic regression analysis of factors using NIHSS subscores revealed three independent factors as predictors of NGT removal after rehabilitation (Table 5): age (OR = 0.907; 95% CI: 0.859–0.957; *p* = 0.000), difference in the FMM score after 4 weeks of rehabilitation (OR = 1.219; 95% CI: 1.145–1.299; *p* = 0.00), and item 9 of NIHSS (OR = 0.488; 95% CI: 0.252–0.946; *p* = 0.034). Older patients, less motor improvement, and less language improvement could result in limited swallowing functional improvement after rehabilitation. By evaluating only one NIHSS item, prediction model 2 was simpler and faster compared to prediction model 1.

**Table 5:** Prediction Model 2—Multivariate analysis for NGT removal after rehabilitation in PSD patients with NIHSS subscores.

**Independent variables**	**OR (95% CI)**	***P*-value**
Age, years	0.907 (0.859–0.957)	0.000
FMM improvement after rehabilitation	1.219 (1.145–1.299)	0.000
Intubated or tracheostomy	4.658 (0.529–41.023)	0.166
Item of NIHSS 1c improvement after rehabilitation	0.573 (0.092–3.569)	0.551
Item of NIHSS 2 improvement after rehabilitation	0.501 (0.121–2.072)	0.340
Item of NIHSS 9 improvement after rehabilitation	0.488 (0.252–0.946)	0.034
Item of NIHSS 10 improvement after rehabilitation	0.734 (0.201–2.682)	0.640

PSD, poststroke dysphagia; NGT, nasogastric tube; NIHSS, National Institute of Health Stroke Scale; FMM, Fugl-Meyer assessment motor score.

### Evaluation of model performance

**Figure 2:**
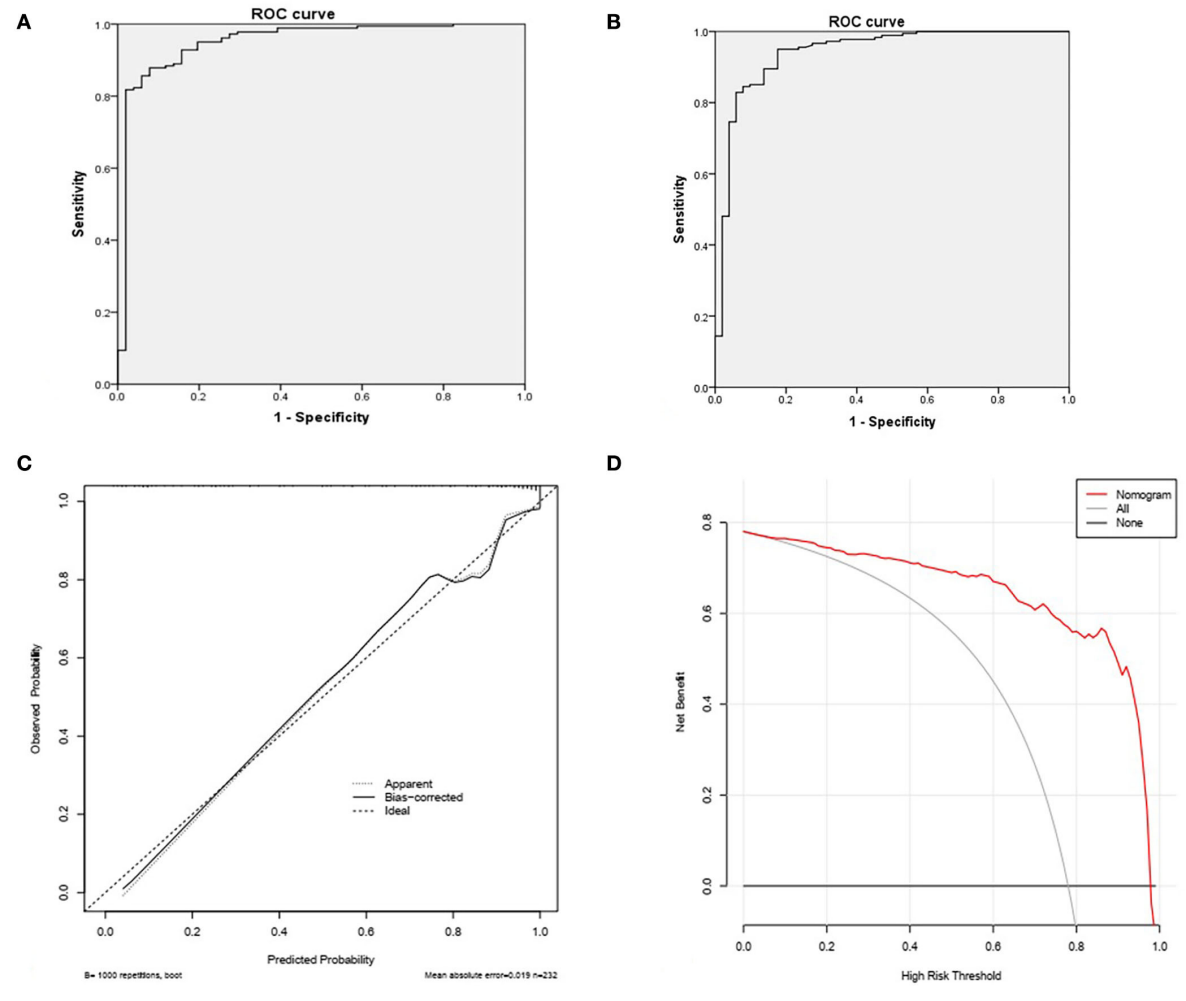
**(A)** The area under ROC curve of Model 1 was 0.950. The sensitivity was 95.0% and the specificity was 82.4%. **(B)** The area under the ROC curve of Model 2 was 0.941. The sensitivity was 92.8% and the specificity was 84.3%. **(C)** The Bootstrap method, repeated sampling 1000 times, was used for validation, and the C-index value was 0.94. **(D)** In the range of 0.01–1, the net benefit rate of the model 2 that predicts NGT removal in PSD patients is >0.

The receiver-operating characteristic (ROC) curve, the calibration curve, and decision curve analysis have been done for the evaluation of model performance (Figure 2). The area under the ROC curve of Model 1 was 0.950. The sensitivity and specificity were 95.0 and 82.4%, respectively. (a) The area under the ROC curve of Model 2 was 0.941. The sensitivity and specificity were 92.8% and 84.3%, respectively. (b) The bootstrap method, which repeated sampling 1,000 times, was used for validation of Model 2 due to more recommendations. The C-index value was 0.94. The calibration curve of the model was close to the ideal curve. (c) In the range of 0.01–1, the net benefit rate of Model 2 that predicted NGT removal in patients with PSD was > 0.

## Discussion

This study was conducted to identify the prognostic factors for NGT removal in patients with prolonged PSD from a large nationally representative dataset. After controlling for confounders, age, motor, and speech functional improvements were significantly associated with NGT removal in patients with PSD. We found no statistically significant correlation between NGT removal in patients with PSD and the following stroke risk factors: arterial hypertension, diabetes mellitus, atrial fibrillation, or hyperlipidemia. This finding was consistent with previous studies.

The correlation between age and NGT removal was statistically significant in our study. Older age was a significant negative predictor of NGT removal, consistent with the results of most previous studies (20, 21). Age-related loss of swallowing muscle mass can manifest as a decrease in the thickness of the tongue, geniohyoid muscle, and pharyngeal wall and an increase in the pharyngeal lumen size. These changes contribute to increased duration of pharyngeal triggering and oral and pharyngeal swallowing, weakened pharyngeal muscle contraction, and deteriorated endurance of swallowing muscles (22, 23). Thus, age-related muscle atrophy may ultimately affect the dysphagia outcome. However, neuroplasticity, which refers to the ability of the central nervous system to alter itself morphologically or functionally, is a major swallowing rehabilitation mechanism. Compared to younger adults, elderly patients recruited their neural networks less efficiently (24).

Dysphagia is managed by a multidisciplinary team, including a physiatrist, a neurologist, an occupational therapist, a ward nurse, and a nutritionist. Therefore, appropriate predictions from swallowing function and other aspects are important. In this study, motor functional improvements from admission to the follow-up in the subacute stage were associated with NGT removal. To the best of our knowledge, this is the first study to investigate the same. Compared to the baseline motor function, motor improvements were more practical clinically as patients admitted for rehabilitation were in different stroke stages, and most of them were not at the state of stroke onset. NIHSS, a systematic, semiquantitative assessment tool for stroke-related neurologic deficits, has been applied to early predict the prognosis of PSD in an acute stage (25). Improvements in NIHSS in patients with PSD in our sample were also associated with NGT removal. The possible reason may be that overall or motor improvement of patient functionality increases brain plasticity, making the patient more capable of relearning how to swallow.

High NIHSS scores are associated with poor outcomes in the first 1 month after discharge (26). Lin et al. (27) suggested that NIHSS items of facial palsy and language/aphasia can be used at the onset of stroke to identify patients with dysphagia at risk of achieving limited improvement. Item 4 of the NIHSS (assessing facial palsy and motor control of the oral cavity) could denote the function of the oral phase in swallowing. Item 9 (assessing language/aphasia) was more likely to be a cognitive factor that affected the interactions between patients and therapists. In our study, in the subacute stage after swallowing rehabilitation, only language/aphasia-associated items could be used to predict the swallowing recovery. Patients with more language/aphasia improvements are likely to resume oral intake because of the capability of understanding, following, and adhering to the guidance of speech therapists. Except for the cognitive factor, improvements in the physiological factors relevant to the dysfunction of swallowing organs could also predict swallowing recovery. Patients with PSD exhibit quantitative differences in the hyoid excursion, laryngeal elevation, tongue base retraction, pharyngeal shortening, and timing of bolus movement (2, 28). Conversely, motor control of the oral cavity (item 4 of the NIHSS) did not predict PSD outcome. Among the NIHSS subitems of dysarthria, facial weakness, and neglect, only severe dysarthria emerged as a significant independent predictor of prolonged dysphagia (12).

The present study had some limitations. First, it was conducted at a single institution. We mitigated these limitations with a relatively large number of patients and a large, nationally representative patient data repository of inpatient rehabilitation. Second, dysphagia severity was not rated at admission. Moderate to severe dysphagia was confirmed in PSD patients with NGT who failed the reliable screening test for dysphagia. Third, the results were mainly restricted to unilateral supratentorial lesion locations, making the research findings less widely used. Differences in resumed oral intake in patients with PSD could be explained by different stroke stages, intervention methods, stroke severities, and sample sizes. Bilateral stroke can lead to the deterioration of swallowing recovery by diminishing compensatory reorganization from the undamaged side of the brain. Additionally, due to the more profound and longer duration of PSD in patients with lateral medullary syndrome than in those with hemispheric stroke, lateral medullary stroke was excluded.

In summary, we established a predictive model for patients with PSD by combining demographic characteristics with functional improvements. Age, motor and overall function improvements, and speech disorders were associated with NGT removal in patients with PSD after 4 weeks of rehabilitation. We believe our results will have practical utility. VFSS was not routinely performed in all patients with PSD. Patients with quick recovery of motor function after rehabilitation passed VFSS and underwent NGT removal with a high possibility. Conversely, rehabilitation is patient-specific, with successful dysphagia therapies implemented in one patient population not necessarily producing the same results in another population. The swallowing rehabilitation program should be adjusted according to the motor recovery to better manage swallowing rehabilitation.

## Data availability statement

The raw data supporting the conclusions of this article will be made available by the authors, without undue reservation.

## Ethics statement

The studies involving human participants were reviewed and approved by China Rehabilitation Research Center Institutional Review Board. The patients/participants provided their written informed consent to participate in this study.

## Author contributions

BL, TZ, JZ, PL, ZW, and SZ are the investigators responsible for project design and protocol writing. TZ, JZ, PL, and ZW contributed to the study background, general design, and study variable definition. SZ and BL participated in sample size calculation and statistical analysis planning, contributed to the preparation of the project, and have read and approved the final manuscript. All authors contributed to the article and approved the submitted version.

## Funding

This study was partially supported by the National Key R&D Program (Adopted Number: 2020YFC2008503).

## Conflict of interest

The authors declare that the research was conducted in the absence of any commercial or financial relationships that could be construed as a potential conflict of interest.

## Publisher's note

All claims expressed in this article are solely those of the authors and do not necessarily represent those of their affiliated organizations, or those of the publisher, the editors and the reviewers. Any product that may be evaluated in this article, or claim that may be made by its manufacturer, is not guaranteed or endorsed by the publisher.
